# A Hemangiopericytoma in the External Auditory Canal: A Rare Clinical Presentation and Management

**DOI:** 10.7759/cureus.68676

**Published:** 2024-09-04

**Authors:** Vaibhavi Patil, Prasad Deshmukh, Sagar S Gaurkar, Ayushi Ghosh Moulic, Jasleen Kaur

**Affiliations:** 1 Otolaryngology - Head and Neck Surgery, Jawaharlal Nehru Medical College, Datta Meghe Institute of Higher Education & Research, Wardha, IND

**Keywords:** case report, external auditory canal, hemangiopericytoma, otolaryngology, radiotherapy, rare tumor, surgery

## Abstract

Hemangiopericytomas (HPCs) are rare vascular tumors originating from pericytes, with a predilection for the musculoskeletal system and occasional occurrence in the head and neck region. HPCs arising in the external auditory canal (EAC) are exceptionally rare, making their diagnosis and management a clinical challenge. A 71-year-old male presented with a six-month history of a painless, progressively enlarging mass in his right EAC, accompanied by tinnitus and hearing loss. Physical examination revealed a mobile, reddish mass in the concha of the left auricle, nearly occluding the EAC. Contrast-enhanced computed tomography of the temporal bone demonstrated a heterogeneously enhancing mass with erosion of adjacent structures. Histopathological examination and immunohistochemistry confirmed the diagnosis of an HPC. The tumor was surgically excised, and the patient underwent adjuvant radiotherapy. Over a two-year follow-up period, no recurrence was observed. This case highlights the rarity of HPCs in the EAC and underscores the importance of considering this diagnosis in patients presenting with atypical EAC masses. A multidisciplinary approach, including surgical excision and radiotherapy, is crucial for achieving favorable outcomes and reducing the risk of recurrence. Long-term follow-up is essential due to the potential for late recurrence.

## Introduction

Hemangiopericytomas (HPCs) are rare vascular tumours originating from pericytes which are contractile cells found around capillaries and venules. First described by Stout and Murray in 1942, HPCs are characterised by their propensity to occur in a wide range of anatomical sites, with the most common locations being the lower extremities, pelvis, and retroperitoneum [1]. However, their occurrence in the head and neck region is relatively uncommon, accounting for approximately 15% of all cases, with even fewer reported in the external auditory canal (EAC) [2,3]. The presentation of HPCs in the EAC is exceedingly rare, with only a handful of cases documented in the literature. These tumours are known for their unpredictable clinical behaviour, ranging from indolent growth to aggressive local invasion and distant metastasis. The rarity of HPCs in the EAC poses significant challenges in diagnosis and management, often leading to delays in appropriate treatment [4].

The diagnosis of HPCs relies heavily on histopathological examination, where the characteristic "patternless pattern" of spindle cells and numerous thin-walled branching vessels, known as "staghorn vessels," are observed [5]. Immunohistochemical staining is also critical, with HPCs typically showing positivity for markers such as CD34, vimentin, CD99, and Bcl-2 and negativity for S100, which helps differentiate it from other spindle cell tumours [6,7]. Given the potential for local aggressiveness and recurrence, complete surgical excision with negative margins remains the cornerstone of treatment for HPC. Adjuvant radiotherapy is often employed in cases with a high risk of recurrence or when complete resection is not feasible [8]. Due to the rarity of this tumour, especially in the EAC, this case report aims to contribute to the existing literature by documenting the clinical presentation, diagnostic process, and successful management of HPCs in this unusual location.

## Case presentation

**Figure 1:**
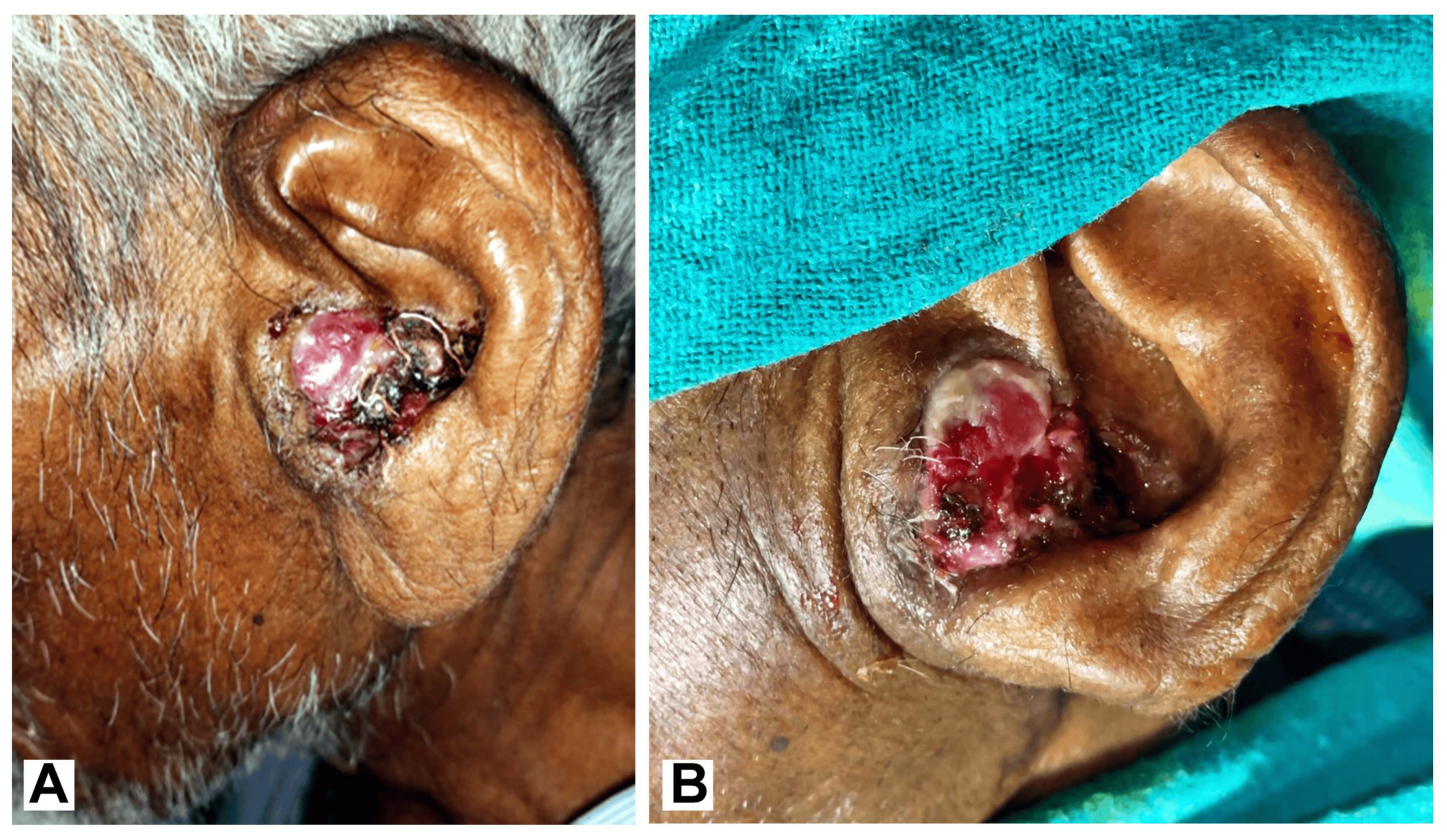
(A-B) The left ear examination was notable for a bulge in the posterior wall of the EAC, leading to near-total closure of the canal, raising concerns about possible underlying malignancy or aggressive benign pathology EAC: External auditory canal

A 71-year-old male presented to the otorhinolaryngology clinic with a six-month history of a painless, progressively enlarging mass in his right EAC. The patient also reported associated tinnitus and progressive hearing loss in the affected ear. His medical history was significant for occasional ear discharge from the left ear since childhood, but no other relevant systemic or otologic conditions were noted. Upon physical examination, a mobile, reddish, soft tissue mass was observed in the concha of the left auricle. The mass extended from the root of the helix to the antitragus, nearly occluding the EAC. The examination did not reveal any cranial nerve involvement. The left ear examination was notable for a bulge in the posterior wall of the EAC, leading to near-total closure of the canal, raising concerns about possible underlying malignancy or aggressive benign pathology (Figure 1).

**Figure 2:**
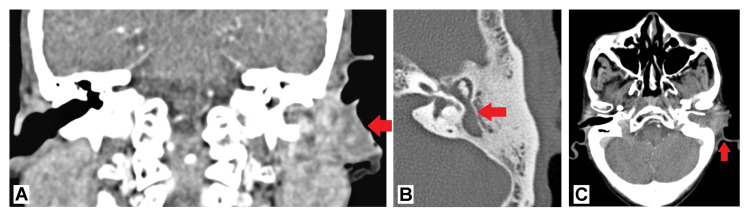
(A) An ill-defined, heterogeneously enhancing soft tissue mass with hypodense areas occupies the left external auditory canal, involving the tympanic membrane. The crux of the helix and tragus are indistinct, with evidence of underlying bony erosion. (B-C) A soft tissue mass is observed in the left external auditory canal

A contrast-enhanced computed tomography (CT) scan of the temporal bone was performed, which revealed a heterogeneously enhancing soft tissue mass within the left EAC. The mass was causing erosion of the adjacent structures, including the left tympanic membrane and the crux of the helix. Additional findings included soft tissue opacification within the middle ear, mastoid sclerosis, loss of mastoid air cell pneumatisation, and disruption of the middle ear ossicles. These imaging findings suggested a locally aggressive process (Figure 2).

**Figure 3:**
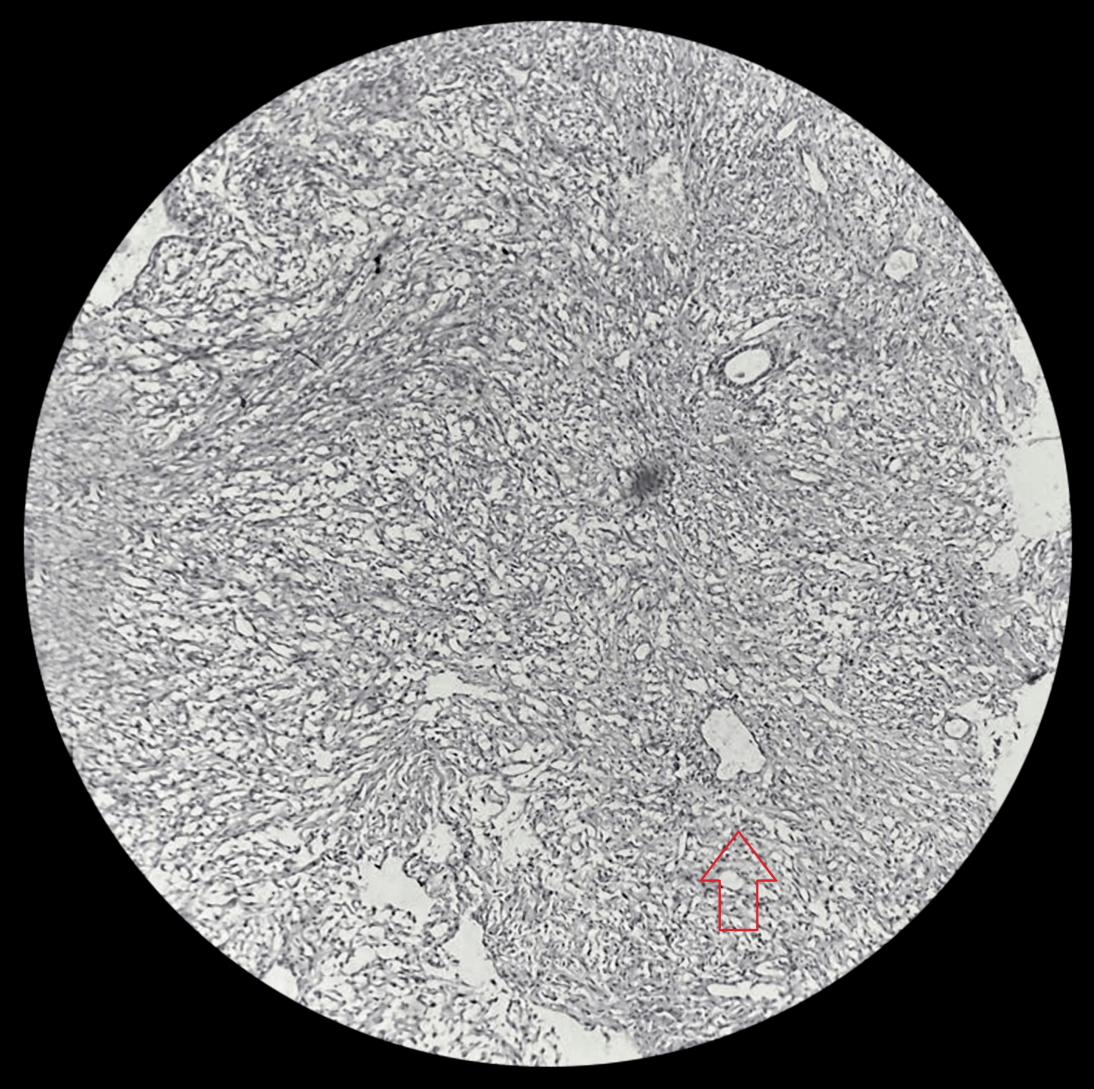
Postoperative histopathological analysis confirmed the diagnosis of the hemangiopericytoma

A mass biopsy was obtained, and histopathological examination revealed a diagnosis of the HPC, characterized by spindle cells arranged in a 'patternless pattern' separated by collagen bands. Immunohistochemistry confirmed the diagnosis, showing positive staining for CD34, vimentin, CD99, and Bcl-2 and negative staining for S100 (Figure 3). Given the patient’s age, symptomatology, and the desire for definitive treatment, surgical excision was pursued. The tumor was excised entirely, measuring approximately 2 cm x 2.5 cm. Postoperative histopathological analysis confirmed the diagnosis of the HPC. Following surgery, the patient underwent adjuvant radiotherapy to minimize the risk of recurrence.

The patient was closely followed for two years postoperatively, when he showed no signs of recurrence. He experienced significant improvement in hearing and a resolution of tinnitus, with no further complications observed. This case highlights the rarity of the HPC in the EAC and underscores the importance of considering this diagnosis when evaluating atypical lesions in this region. The successful management of this patient through surgery and radiotherapy demonstrates the effectiveness of a comprehensive approach in treating such rare and potentially aggressive tumours.

## Discussion

HPCs are rare vascular tumours originating from Zimmermann's pericytes, which are contractile cells located around capillaries and venules. These tumours can arise throughout the body, most commonly in the musculoskeletal system and less frequently in the head and neck region. The occurrence of HPCs in the EAC is infrequent, with only a few cases reported in the literature, making this case noteworthy.

HPCs in the EAC present a diagnostic challenge due to their nonspecific clinical presentation. Patients typically present with symptoms such as a painless mass, hearing loss, and sometimes tinnitus, as seen in our patients. These symptoms overlap with those of more common EAC lesions, such as ceruminous gland tumours, squamous cell carcinoma, or cholesteatomas. It is imperative to consider HPCs in the differential diagnosis when encountering an unusual mass in this region [9].

The diagnosis of HPCs relies heavily on histopathological examination and immunohistochemical staining. Histologically, HPCs are characterised by a "patternless pattern" of spindle cells separated by thin-walled, branching blood vessels. Immunohistochemically, HPCs typically express markers such as CD34, vimentin, CD99, and Bcl-2 while being negative for S100, which helps differentiate them from other spindle cell neoplasms like schwannomas or meningiomas [10]. In this case, the diagnosis of HPCs was confirmed by the presence of these characteristic features.

The management of HPCs generally involves complete surgical excision, which is considered the treatment of choice. In cases where complete resection is not feasible or when the tumour shows aggressive features, adjuvant radiotherapy may be employed to reduce the risk of local recurrence [11]. Our patient underwent total excision of the tumour followed by radiotherapy, which has proven to be an effective treatment strategy. Over the two-year follow-up period, no signs of recurrence were observed, underscoring the importance of a comprehensive approach in managing such cases.

Despite successful treatment, long-term follow-up is essential for patients with HPCs due to the potential for local recurrence and, albeit rare, metastatic spread. The literature suggests that HPCs can recur many years after the initial treatment, necessitating vigilant long-term monitoring [4]. Our patient's favourable outcome, with no recurrence over two years, aligns with reports suggesting that early and complete excision, combined with radiotherapy, offers a good prognosis.

## Conclusions

This case of an HPC in the EAC underscores the importance of considering rare tumours in the differential diagnosis of atypical masses in the ear. Despite its rarity, the HPC can present with nonspecific symptoms that mimic more common ear pathologies, making accurate diagnosis essential for appropriate management. The successful treatment of this patient through complete surgical excision followed by radiotherapy, coupled with vigilant follow-up, resulted in a favourable outcome with no recurrence observed over two years. This case highlights the value of a multidisciplinary approach in managing rare and potentially aggressive tumours, emphasising the need for early detection and comprehensive treatment to achieve optimal patient outcomes.
